# Active surveillance of atypical cartilaginous tumours of bone: short term quality of life measurements

**DOI:** 10.1186/s13018-023-03694-9

**Published:** 2023-03-16

**Authors:** Claudia Deckers, Nander T. van Zeijl, Miranda L. van Hooff, Petra I. Veldman-Goossen, H. W. Bart Schreuder, Edwin F. Dierselhuis, Ingrid C. M. van der Geest

**Affiliations:** Department of Orthopaedic Surgery, Radboud University Medical Centre, Nijmegen, The Netherlands

**Keywords:** Chondrosarcoma, Watchful waiting, Bone neoplasms, Quality of life

## Abstract

**Background:**

In the recent years active surveillance has been introduced for atypical cartilaginous tumours (ACT). This is the first study on the impact of this new treatment approach on patients’ quality-of-life. We evaluated general health-related quality of life (HRQL) in patients diagnosed with enchondroma or ACT.

**Methods:**

In this prospective study, patients recently diagnosed with enchondroma and ACT of the long bones were asked to participate. Health-related quality of life (HRQL) was assessed at diagnosis and at six month follow-up, using the 36-item Short Form Health Survey (SF-36) and Numeric Pain Rating Scale (NRS). HRQL of the active surveillance group was compared to the Dutch population and a Dutch sample with locoregional cancer.

**Results:**

In total, 45 patients were included in the study, of which four patients underwent curettage and cryosurgery, 41 patients were under active surveillance. The HRQL of the active surveillance group seemed lower compared to the Dutch population, but similar to patients suffering locoregional cancers. No comparison between the surgery and the active surveillance group could be made. In the active surveillance group no statistical difference was found between baseline and six months follow-up regarding HRQL and pain during rest and activities.

**Conclusion:**

Patients diagnosed with non-malignant chondroid tumours have lower HRQL compared to the healthy population. Active surveillance had no adverse effect on patients well-being, after six months active surveillance the HRQL remained unchanged. Interestingly, in our study no impact on mental health was seen, implicating that diagnosed but untreated chondroid tumours do not seem to influence patients anxiety.

## Background

Enchondroma and atypical cartilaginous tumours are primary bone tumours consisting of cartilaginous matrix [1]. Over the last decades, an increase in incidence of enchondroma and atypical cartilaginous tumours (ACT) has been observed, probably due to the increased detection rate of asymptomatic tumours [2, 3]. They are nowadays the most common primary bone tumours [2, 3].

Enchondroma are benign inactive bone tumours and patients with enchondroma are commonly discharged from follow-up [1]. ACT is a locally aggressive tumour located in the appendicular skeleton. For patients with ACT, intralesional surgery with local adjuvant therapy is recommended [1]. Morbidity of this treatment is low but complications such as postoperative fractures, infection, and local recurrence do occur [4, 5]. The necessity of surgical treatment for ACT lesions has been subject of debate the last years for several reasons [6]. First, in most patients incidental found cartilaginous tumours are asymptomatic and development of clinical manifestations over time is rare [7, 8]. Secondly, with the current standards for treatment of cartilaginous tumours in mind, the diagnostic challenge to differentiate enchondroma from ACT due to the numerous similarities on imaging [9], results in a therapeutic dilemma.

ACT are nowadays defined as local aggressive tumours, with non-metastatic potential, instead of malignant cartilaginous tumours [1], and refraining from surgery might be an attractive treatment strategy [3, 7, 10, 11]. In the recent years active surveillance has been introduced for ACT to prevent over-treatment. In active surveillance regular radiologic follow-up is performed to monitor the tumour [7, 11]. If the tumour shows any signs of progression (e.g. growth or pain) the tumour can still be removed by performing intralesional surgery with local adjuvant therapy. Omlor et al. showed that surgery of enchondroma and ACT did not prove superior compared to conservative clinical and radiological observation, whereas the conservative approach showed a significantly better functional outcome compared to the surgical treatment [10].

The impact of this new conservative treatment approach on the patient’s quality-of-life should also be considered. It could be argued that the burden of diagnosed but untreated disease outweighs the benefit of withholding surgery. Active surveillance might be new for non-malignant cartilaginous tumours (i.e., enchondroma and ACT) but in several other fields in oncology it is already a common treatment method. Studies performed in the field of prostate cancer have shown that active surveillance has no major adverse effect on patients wellbeing [12, 13].

To the best of our knowledge, no evidence exists on how active surveillance for patients with chondroid bone tumours impacts patients’ quality of life. The objective of this study is to measure the impact of this new treatment approach on the patient’s quality-of-life. In this study we evaluate general health-related quality of life (HRQL) in patients diagnosed with non-malignant chondroid tumours.

## Patient and methods

This prospective cohort study was designed as a single-centre observational study and follows the STROBE guidelines [14]. From April 2018 till July 2020, all adult patients referred to our orthopaedic oncology outpatient clinic with a recently diagnosed enchondroma in the long bones or ACT were invited to participate in this quality-of-life study. Exclusion criteria were patients with suspected high grade chondrosarcoma on imaging (e.g. cortical destruction, soft tissue extension), Ollier or Maffucci disease, tumours localized juxtacortical or in the axial skeleton, because these tumours have a more aggressive biological behaviour [1]. Patients who underwent previous treatment elsewhere were also excluded. For the purpose of this study patients were excluded when they did not complete both the questionnaires at baseline and at six months follow-up assessment.

This study has been reviewed and approved by the ethics committee of our hospital on the basis of the Dutch Code of conduct for health research, the Dutch Code of conduct for responsible use, the Dutch Personal Data Protection Act and the Medical Treatment Agreement Act. Exemption was obtained as the Medical Research Involving Human Subjects Act does not apply for this study. Written informed consent was obtained from all patients included in this study.

During the first consultation, diagnosis and treatment options (active surveillance versus intralesional surgery with local adjuvant treatment) were discussed with the patient by the orthopaedic oncologist. A digital decision aid developed by our team was available for patients to provide them with up-to-date information on the diagnosis and current treatment options [15]. After one week, wherein the patient could have studied the digital information, and discussed the options with relatives, the patient was called by the nurse practitioner or the orthopaedic oncologist to decide on the treatment. No extra training in shared decision making techniques were followed. All patients that participated in this study used the digital decision aid, majority felt that it supported in their decision making.

If patients chose surgery, they were treated with curettage and cryosurgery (CC-group), and depending on the bone defect osteosynthesis (plating) was used [16]. Postoperative follow-up consultations were planned at six months and at 18 months after the treatment. If patients chose for active surveillance (AS-group) then radiologic follow-up, preferably magnetic resonance imaging (MRI), was performed at six months and at 18 months after diagnosis [7]. Depending on radiologic criteria and clinical manifestations, radiologic follow-up was continued every year or every two years. When tumour show progression during follow-up without development of malignant characteristics (e.g., cortical destruction, soft tissue extension), next follow-up MRI is recommended within one year. When follow-up shows no changes, a biennial MRI is recommended [17].

Patients included in the current quality of life study were sent digital questionnaires after the first consultation and at six months after diagnosis, irrespective of the chosen treatment. The questionnaire was sent at six months after diagnosis as this is the first follow-up for both the surgical group as the active surveillance group.

Demographic (age, sex), clinical (referral, tumour size, location) treatment (surgery, surveillance) and outcomes data were collected as part of routine follow-up.

### Outcome measures

Patient-reported health-related quality of life (HRQL) was measured using the Dutch version of the Short Form-36 (SF-36), a general health assessment instrument [18]. We choose to use a generic health questionnaire as both enchondroma and ACT are mostly incidental findings and only rarely cause complaints. The SF-36 is a widely used generic health questionnaire and has shown good reproducibility in the Dutch population [18].

The SF-36 measures consists of eight subscales that can be combined into a physical component summary (PCS) and a mental component summary (MCS) scale. The eight subscales include physical functioning (PF), body pain (BP), general health perceptions (GH), mental health (MH), social functioning (SF), vitality (VT), and role limitations due to physical (RP) and emotional problems (RE). To create the Mental Component Summary (MCS) and Physical Component Summary (PCS) answers were combined using the method as described by Ware et al. [19]. Scores of component scales and subscales range from 0 to 100, with higher scores indicating better HRQL. The SF-36 has been translated and validated to Dutch, norm values are available for the Dutch population [18].

In this study we compared the SF-36 scores of the active surveillance group with norm groups of the Dutch population and a Dutch sample with locoregional cancer [18].

In addition, the Numeric Rating Scale (NRS) for pain, ranging from zero (no pain) to 10 (worst pain imaginable), was measured. The NRS was used for two questions. Patients were asked if they experienced any pain during rest (NRSr) and if they experienced any pain during activities (NRSa) and they rated it accordingly.

### Statistical analyses

Baseline patient characteristics were explored for both the study group and the group who did not respond to the follow-up questionnaires, (non-respondents) to exclude any significant differences. Patient who did not complete both questionnaires (baseline and six months) were excluded from further analyses. All continuous variables were visually inspected and tested for normality by the Shapiro–Wilk test. For both AS and CC-group continuous variables are described and presented as the mean and standard deviation (SD) or as the median and inter quartile range (IQR), depending on normality. Categorical variables were described as counts and percentages and compared using the Fisher exact test. To compare non parametric continuous variables of the AS-group between baseline and six months follow-up the Wilcoxon signed rank test was used. Since only four people were included in the CC-group their results of the baseline and six months follow-up were only described.

A sensitivity analyses was performed to analyse if having had the follow-up MRI impacted HRQL after six months. The SF-36 results at six months were compared using the Wilcoxon signed rank test of these patients that already underwent the follow-up MRI, when completing the six months questionnaire, and those patients that were awaiting there follow-up MRI.

All *p*-values were tested two-sided, and a *p*-value of < 0.05 was considered statistically significant.

**Fig. 1:**
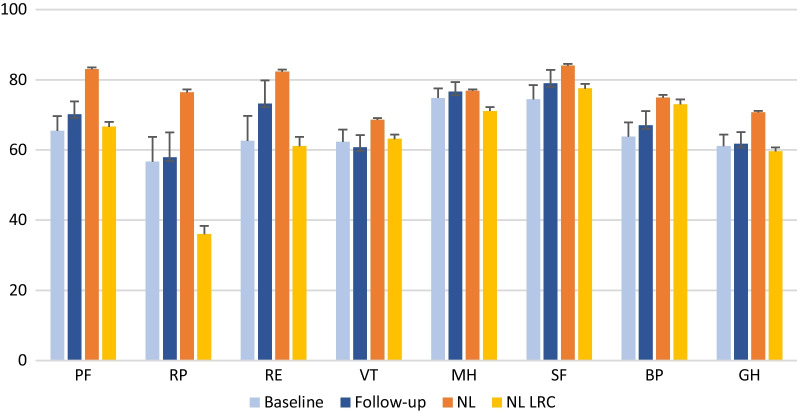
Comparison of SF-36 subscales. Active surveillance group (*n* = 41) at baseline and follow-up compared to Dutch population (NL, *n* = 1742), Dutch population suffering local regional cancer (LRC, *n* = 286). Bars reflect mean SF-36 scores and standard error of the mean (SE). *PF* Physical function, *RP* Role limitations due to physical, and *RE* Emotional problems, *VT* Vitality, *MH* Mental health, *SF* Social functioning, *BP* Body pain, *GH* General health

Despite our data being non parametric and to compare results with the Dutch norm groups the results of the current study are presented in Fig. 1 as mean and standard error (SE). We visually compared the results.

All data was collected from the hospital digital charts by January 2021 and stored in a secured environment by using Castor EDC (Castor electronic data capture, Amsterdam, the Netherlands). Statistical analyses were performed using SPSS 25 software (IBM SPSS Statistics for Windows, Version 25 IBM Corp., Armonk, NY, USA.) and R version 3.6.2 (R Foundation for Statistical Computing, Vienna, Austria).

## Results

Seventy patients were asked to participate in this study, two of whom did not receive the questionnaires. Of the 68 patients participating, 23 patients were excluded as they did not complete the SF-36 questionnaire at both measurement times. In total, 45 patients (13 male, 32 female) that reported HRQL outcomes, both at baseline as well as after six months of follow-up (response rate 66.2%), were included in the analyses. At baseline, patient characteristics and tumour characteristics for both the study group (*n* = 45) and the non-respondents (*n* = 23) did not differ (Table 1).

**Table 1:** Patient characteristics

	Study group (*n* = 45) N (%)	Non-respondents (*n* = 23) N (%)	*p*-value
Mean age ± SD	52 ± 10.9	54 ± 12.8	.32
*Gender*			.55
Male	13 (28.9)	7 (30.4)	
Female	32 (71.1)	16 (69.6)	
*Treatment*			.44
Surveillance	41 (91.1)	20 (87)	
Surgery	4 (8.9)	3 (13)	
*Lesion location*			.56
Upper extremities	17 (37.8)	9 (39.1)	
Lower extremities	28 (62.2)	14 (60.9)	
*Lesion size*			.65
0 – 2 cm	5 (11.1)	2 (8.7)	
2 – 5 cm	22 (48.9)	13 (56.5)	
5 – 10 cm	16 (35.6)	7 (30.4)	
10 – 15 cm	2 (4.4)	1 (4.3)	
*Referral indication*			.18
Incidental	39 (86.7)	17 (73.9)	
Pain	6 (13.3)	4 (17.4)	
Second opinion	0 (0)	1 (4.3)	
Unknown	0 (0)	1 (4.3)	

*p*-values were derived by a paired samples *t*-test for the variable age, for all the other variables fisher exact test was used

### Health-related quality of life changes

In Table 2 the results of the SF-36 and NRS questionnaires at baseline and follow-up are shown. At both baseline and follow-up, the surgery group (*n* = 4) seemed to have higher pain scores compared to the active surveillance group (*n* = 41) and lower scores on the following SF-36 subscales; body pain and role limitations due to physical function. On the remaining SF-36 subscales the surgery (curettage and cryosurgery; CC) group and the active surveillance (AS) group scored similar results. HRQL changes between baseline and follow-up were tested for the active surveillance group. No comparison between the AS and CC-group was made as only four patients underwent surgery. No significant changes in any of the eight SF-36 subscales (physical functioning, physical role, emotional role, vitality, mental health, social functioning, bodily pain, general health) were seen when baseline results were compared to six months follow-up. Nor did any significant changes occur in the physical and mental health component scores over time.

**Table 2:** HRQL outcomes as measured by SF-36 and NRS compared between baseline and six months follow-up

	Baseline		Follow-up		*p*-value
	AS (*n* = 41)	CC (*n* = 4)	AS (*n* = 41)	CC (*n* = 4)	AS
PF	70 [43–90]	67.5 [61–85]	75 [55–90]	80 [24–88]	.17
RP	75 [0–100]	0 [0–75]	75 [0–100]	0 [0–75]	.88
RE	100 [0–100]	100 [100–100]	100 [33–100]	100 [100–100]	.15
VT	70 [45–83]	65 [45–78]	65 [40–78]	65 [61–69]	.31
MH	80 [68–86]	74 [69–79]	80 [68–90]	80 [73–93]	.55
SF	75 [63–100]	75 [53–97]	87.5 [63–100]	68.8 [16–84]	.17
BP	67.5 [45–85]	50 [45–64]	67.5 [45–90]	51.3 [19–73]	.38
GH	60 [43–80]	60 [56–60]	65 [50–78]	72.5 [70–75]	.82
PCS	44.6 [32–54]	32.9 [32–44]	43.4 [36–52]	36.9 [21–46]	.67
MCS	54.8 [39–59]	56.6 [52–59]	54.2 [45–60]	56.8 [55–59]	.59
NRSr	1.0 [0–5]	3.0 [2–6]	1.0 [0–5]	2.5 [0–7]	.35
NRSa	3.0 [0–6]	6.5 [3–7]	2.0 [0–6]	6.0 [2–8]	.87

Values are presented as median [IQR]. *P*-values were derived by performing Wilcoxon signed rank test

*AS* Active surveillance, *CC* Curettage and cryosurgery, *PF* Physical function, *RP* Role limitations due to physical. And *RE* Emtional problems, *VT* Vitality, *MH* Mental health, *SF* Social functioning, *BP* Body pain, *GH* General health, *PCS* Physical component summary, MCS Mental component summary. *NRSr* NRS score in rest, *NRSa* NRS score during activity

Fourteen out of the 41 patients (34%) in the active surveillance group had their follow-up MRI before they completed the second questionnaire. To analyse if having had the follow-up MRI impacted HRQL after six months, additional Wilcoxon Signed Rank analyses were performed. No significant differences in any of the eight SF-36 subscales or component scores at six months follow-up were found when the 14 patients who already had their follow-up MRI are compared with the 27 patients still awaiting follow-up MRI.

In addition to the SF-36, NRS pain scores in rest and during activity were measured at baseline and follow-up. The surgery group reported higher pain scores (NRSr median 3.0 [2–6]; NRSa median 6.5, IQR [3–7]), interestingly they did not decrease at six months follow-up. All four patients who went for surgery had their surgery within the first six months after diagnosis. No significant changes between baseline and six month follow-up in pain scores were detected for the surveillance group.

### HRQL of the active surveillance (AS) group compared to Dutch norms

As depicted in Fig. 1, subscales of the SF-36 at baseline and follow-up of the active surveillance group were compared to data of a healthy Dutch norm group. The active surveillance group showed comparable scores in mental health but lower results on most subscales.

Visual comparison of the current study results with a Dutch sample with local regional cancer (LRC) shows similar results on most subscales (PF, RE, VT, MH, SF, GH). The LRC group scored lower on role limitations due to physical problems (mean 36, SE 2.4) but scored slightly higher on the subscale body pain (mean 73, SE 1.4).

## Discussion

In this study we evaluated the health-related quality of life (HRQL) of patients with chondroid tumours, without malignant characteristics, located in the long bones. This is the first study that reports HRQL of patients with chondroid bone tumours under active surveillance. It is important to explore if the burden of diagnosed but untreated disease outweighs the benefit of refraining from surgery. HRQL was measured after diagnosis and during follow-up, no changes in HRQL over time were found. Compared to the Dutch population the active surveillance group scored lower on most SF-36 subscales, however results were similar when compared with patients suffering local regional cancers [18].

Of the 45 patients included in this study only four patients opted for surgical treatment whereas the majority, 41 patients, chose active surveillance. The high rate of patients choosing active surveillance might be caused by the available decision aid. It is known that the use of decision aids tends to shift patients preferences towards non-surgical interventions [20]. We were unable to apply statistical methods on the surgical treatment-group (CC-group), as only few patients chose surgery. In the active surveillance-group (AS-group), no significant changes in any of the eight SF-36 subscales, SF-36 summary scores, and NRS pain scale could be observed when baseline results were compared with six months follow-up. This indicates that active surveillance has no adverse effect on patients wellbeing on a short time of follow-up, between diagnosis and 6 months later.

Interestingly, the CC-group reported high pain scores preoperatively, which did not decrease several months after surgery (Table 2). These pain complaints might still be explained by the surgery performed due to the relative short follow-up of this study. Omlor et al. reported results after 24 months follow-up and showed that the surgically treated patients with enchondroma or ACT had worse results for pain compared to conservatively treated patients [10]. Surgery is often advised when patients experience pain, however it has not been proven to diminish pain complaints. It should be considered that pain might not be related to the tumour nor the operation but to adjacent pathology instead.

In this study we were not able to compare results between surgery and active surveillance, due to the small surgery group. There were no studies found in literature reporting results of all SF-36 subscales of patients with chondroid tumours treated with intralesional surgery. Van der Geest et al. reported in their study only the physical functioning subscale (mean 58; SD 29), which was significantly lower than the norm scores of the Dutch population and seems lower than our results of the AS-group [21]. Shchelkova et al. reported results of the SF-36 of 22 patients with chondrosarcoma (ACT and grade 2 chondrosarcoma) but it is not reported if these patients were operated on [22]. Since there is little knowledge on HRQL after intralesional surgery of chondroid tumours it is difficult to put our HRQL results of active surveillance in context.

We visually compared the results of the SF-36 subscales of our AS-group with the Dutch population (Fig. 1). HRQL of our study group seemed lower on all subscales with the exception of the subscale mental health (MH). As there are several similarities between the SF-36 mental health subscale and typical depression and anxiety screening questionnaires we presume that diagnosed but untreated chondroid tumours do not seem to influence patients anxiety. In addition, the mental health component score (MCS) at six months follow-up assessment was 57 (IQR 52–59) whereas the threshold for mental disorder is a MCS of ≤ 38 [23].

On the other subscales the AS-group seems to score lower, implicating that diagnosis and treatment of non-malignant chondroid tumours impacts HRQL negatively. Visual comparison showed the greatest difference on the subscales physical function (PF), role limitations due to physical problems (RP), role limitations due to emotional problems (RE), body pain (BP), and general health (GH). The lower scores on the subscales PF, RP, and BP were unexpected, as 87% of the included tumours in our study are incidental findings on imaging and therefore assumed to be asymptomatic. Adjacent pathology (e.g., osteoarthritis or trauma), the initial reason for performing imaging, might be the reason for the lower HRQL scores. For example, our results on the subscales PF, RP, BP and GH are comparable to the HRQL results of patients suffering osteoarthritis [24]. The SF-36 questionnaire is a general health assessment instrument and comorbidities should have been taken into account. In this study, 87% of the patients suffered from health problems leading to having imaging performed. We did not have information on patients comorbidities. For this reason, we cannot conclude that the lower HRQL of the patients in this study was solely caused by the diagnosis and treatment of chondroid tumours.

In addition to the healthy Dutch population, we also visually compared our results with patients suffering from local regional cancer (LRC). Except from the subscale bodily pain (BP) the LRC-group scored similar or lower compared to the AS-group. Visual comparison showed the greatest difference on the subscale role limitations due to physical problems (RP). This could be explained by the chemotherapy and/or radiotherapy treatment received by the patients in the LRC-group. The AS-group scored lower than the Dutch population on the subscale role limitations due to emotional problems (RE). Compared to the LRC-group, results on the subscale RE were similar. However, as shown in Fig. 1, an increased trend is seen in the subscale RE after six months follow-up, reporting higher results than the LRC-group. Presumably, a longer follow-up could potentially show a significant increase in the subscale RE as patients might be reassured of the non-malignant character of the tumour when the tumour remains stable during follow-up.

To our knowledge, this is the first study performed evaluating the HRQL of patients with enchondroma or ACT who underwent active surveillance, hence comparison with similar studies could not be made. Therefore, we broadened our view and searched for published results in related oncological fields where active surveillance is more common. Studies on chronic lymphocytic leukaemia and prostate cancer show contradictory results: in most cases those on active surveillance reported higher HRQL compared with those undergoing treatment but a few studies showed that those on active surveillance experienced greater anxiety and depression [25]. As reported in the systematic review of Kim et al., including 73 studies, patients with chronic lymphocytic leukaemia or prostate cancer were dissatisfied with the information received [25]. The experienced lack of information could be an explanation for the increased anxiety and depression. It is known that education and communication alleviates anxiety and improves the sense of patients control of the situation [26]. We have provided the patients in this study with a digital decision aid which could explain why we did not find any impact of active surveillance on the mental health subscale. Decision aids improve people’s knowledge and reduce the decisional conflict [20].

Several limitations should be discussed. First, this study lacked data on employment status, socioeconomic status, marital status, and education level, all factors that could influence HRQL. The impact of comorbidities should also have been taken into account. It is common that chondroid tumours are incidental findings on imaging performed for adjacent pathology, such as osteoarthritis. It is known that musculoskeletal disease have a substantial negative impact on HRQL [24]. Since we did not take the impact of comorbidities into account this might have negatively impacted the HRQL reported in our study. We hypothesize that the reported HRQL of patients with chondroid tumours would have been higher when patients suffering from musculoskeletal diseases such as osteoarthritis were excluded from our study sample. The HRQL in this study can therefore be influenced, either positively or negatively, by different potentially influencing factors that were not measured.

Second, this study was limited by the skewedness of the data. To be able to compare the results of the SF-36 questionnaires with the Dutch population the mean and standard error were reported despite the skewed distribution of our data. As such, no statistical methods could be applied to compare the data and only visually inspection was performed. In addition, no comparison between the AS and CC-group could be made as only four people in this study opted for surgery. We don’t know if surgery might have increased the patients HRQL at follow-up. Therefore we cannot fully answer the question if the burden of diagnosed but untreated disease outweighs the benefit of withholding surgery.

Third, this study reported the results of short-term HRQL and future research using long-term HRQL is recommended. Active surveillance for chondroid tumours without malignant characteristics is becoming more popular [7, 8, 10, 11], as such report of the current first short-term HRQL results is acceptable.

In conclusion, patients diagnosed with non-malignant chondroid tumours have lower HRQL compared to the healthy population. Interestingly, in our short-term study no impact on mental health was seen, implicating that diagnosed but untreated chondroid tumours do not seem to influence patients anxiety. Furthermore, active surveillance had no adverse effect on patients wellbeing, no statistical difference was found between baseline and six months follow-up regarding HRQL and pain during rest and activities. It is important that orthopaedic oncologist are aware of the lower HRQL of patients diagnosed with non-malignant chondroid tumours and educate their patients to alleviate anxiety.

## Data Availability

The datasets used and/or analysed during the current study are available from the corresponding author on reasonable request.

## Abbreviations

ACT: Atypical cartilaginous tumours
LRC: Local regional cancer
CC: Curettage and cryosurgery
AS: Active surveillance
MRI: Magnetic resonance imaging
HRQL: Health related quality of life
NRS: Numeric rating scale
SF-36: Short form health survey
PF: Physical function
RP: Role limitations due to physical problems
RE: Role limitations due to physical problems
VT: Vitality
MH: Mental health
SF: Social functioning
BP: Body pain
GH: General health
PCS: Physical component summary
MCS: Mental component summary
